# Early environmental risks and the developmental dynamics of internalizing and externalizing problems from birth to adolescence

**DOI:** 10.1007/s00787-025-02887-3

**Published:** 2025-11-06

**Authors:** Kehui Wu, Francesca Zecchinato, Canghai Guan, Hongyi Sun

**Affiliations:** 1 Department of Sociology, Faculty of Social Sciences, University of Macau, Macau, China; 2 NIHR ARC Wessex Mental Health Hub, School of Health Sciences, University of Southampton, Southampton, UK; 3 Department of National Law and Basic Theory, Law School, Liaoning University, Shenyang, China; 4 Centre for Innovation in Mental Health, School of Psychology, University of Southampton, Southampton, UK

**Keywords:** Early environmental risks, Internalizing problems, Externalizing problems, Comorbidity

## Abstract

Internalizing and externalizing problems often co-exist throughout an individual’s development, shaped by a shared set of early environmental risks. However, most existing studies focused on discrete developmental periods, limiting understanding of how the negative impact of early-life adversity on mental health varies with age. Using data from 7,377 participants across seven waves of the UK Millennium Cohort Study (MCS), the current study investigated the bidirectional associations between internalizing and externalizing problems from ages 5 to 17, as well as the long-term impact of early environmental risks (i.e., prenatal influences, neonatal factors, maternal mental health, harsh parenting, and socioeconomic status) on the two symptom domains across time. Random-intercept cross-lagged panel analyses indicated that individuals with higher internalizing symptoms tended to report higher externalizing symptoms, with both symptom domains showing high stability over time. A significant positive bidirectional relationship between internalizing and externalizing problems was found from childhood to early adolescence. However, this pattern diverged in late adolescence: internalizing problems at age 14 no longer predict externalizing problems at age 17, whereas externalizing problems negatively predict subsequent internalizing symptoms. Early environmental risk factors significantly predicted both internalizing and externalizing problems from ages 5 to 14, with the effects generally decreasing with age, except for an increase at age 11. Gender differences were also observed in both the bidirectional relationships between the symptom domains and the long-term impact of early environmental risks. Findings underscore the enduring impact of early adversity on adolescent mental health and highlight the complex, evolving interaction between internalizing and externalizing problems. The study offers critical insights for early, sustained interventions that address multiple risks and adapt to adolescents’ changing mental health needs over time.

**Supplementary Information:**

The online version contains supplementary material available at 10.1007/s00787-025-02887-3.

## Introduction

Mental ill-health often emerges during the early developmental period [1] and, if left untreated or unaddressed, tends to crystallize into psychopathological presentations, such as depressive disorders and behavioral difficulties, in later childhood and adolescence, with long-lasting pervasive impacts on individuals’ lives [2–5]. Mental health difficulties in children and young people are typically categorized into two domains: internalizing and externalizing problems [6]. Internalizing problems are mostly inner-directed, resulting in disturbances in emotional states such as anxiety and depression [6]. Externalizing problems refer to outer-directed behaviors that cause discomfort and conflict in the external environment, encompassing observable behaviors, such as conflict with others, aggression, rule-breaking, and behavioral dysregulation, including hyperactivity [6, 7]. Internalizing and externalizing problems in childhood can be detrimental to individual’s development, leading to widespread negative social and behavioral outcomes such as peer problems, substance abuse, and antisocial behaviors [2, 8, 9]. In addition, both internalizing and externalizing problems are associated with physical illness [10].

Internalizing and externalizing problems often co-exist. Previous literature has framed the co-development of internalizing and externalizing problems over time from a developmental perspective [2, 11]. For example, the failure theory proposes a unidirectional relationship from externalizing to internalizing problems. Specifically, externalizing problems in childhood, such as aggression, rule-breaking, and hyperactivity, often lead to later internalizing problems through a process characterized by experiences of failure in social and educational domains [12]. The rejection and academic failure elevate the risk of children and adolescents experiencing feelings of worthlessness, loneliness, and social withdrawal, which in turn increase the risk of internalizing problems such as anxiety and depression symptoms [12]. Conversely, the acting-out model lends support to the link from internalizing to externalizing problems, suggesting that children who experience emotional distress may manifest these internal struggles as outward behaviors, such as aggression and conflict with others, thereby exhibiting more externalizing problems [13]. Beyond the unidirectional relationships, the adjustment erosion hypothesis proposes a bidirectional relationship between the internalizing and externalizing domains, where difficulties in one domain can erode an individual’s adjustment in the other [14]. According to this hypothesis, children exhibiting either internalizing or externalizing problems tend to experience higher levels of peer rejection, academic failure, and family stress [14]. Such difficulties significantly impair the individual’s overall adjustment, contributing to a cycle of co-occurring problems over time [14, 15]. Collectively, theories and empirical studies have produced inconsistent findings regarding the bidirectional relationships between internalizing and externalizing problems. One possible explanation is that much of this research has focused on specific developmental periods, such as middle childhood or adolescence (e.g., [15, 16]). The narrow focus constrains our understanding of how these difficulties interact and evolve across the broader developmental span. Consequently, research that examines a longer developmental trajectory would offer a more comprehensive understanding of the co-occurrence of these two symptom domains.

A further explanation for the co-occurrence of internalizing and externalizing problems may lie in common underlying mechanisms shaped by shared risk factors, which can simultaneously influence the development and maintenance of both symptom domains [16]. Although the inheritance of genetic risk factors is known to play a role in the development of mental health difficulties in children, a substantial body of evidence shows that a range of environmental factors are associated with both internalizing and externalizing problems in children and young people, even after accounting for genetic risk [17–20]. These include the prenatal and postnatal rearing environment, familial economic and social resources, parental mental health, parenting practices, and the quality of parental relationship [17, 19–23]. A consistent body of evidence indicates that maternal factors, such as maternal smoking during pregnancy, maternal pre- and postnatal mental health (particularly depression and anxiety), are associated with adverse mental health outcomes in children and adolescents [17, 24–27]. Disciplinary parenting practices, particularly harsh or coercive parenting styles, as well as overcontrolling behaviors, are also linked to children’s internalizing and externalizing problems [28–31]. Socioeconomic disadvantages, such as low family income, low maternal education level, and house overcrowding, have been found to be positively associated with mental health problems in childhood as well [18, 24]. Furthermore, neonatal factors, including prematurity (gestational age < 37 weeks), low birth weight, and the absence of breastfeeding, are associated with increased risks of both internalizing and externalizing problems in childhood [32–34]. Specifically, all three neonatal factors are associated with impaired neurological development and increased perinatal complications, including disruptions in brain maturation and connectivity, which may lead to mood disorders and behavioral disorders [34–36]. In particular, breastfeeding promotes early mother-infant bonding, which fosters secure attachment and emotional regulation [37]. On the contrary, children who are not breastfed may therefore miss out on these protective biological and relational benefits, placing them at greater risk of internalizing and externalizing problems [34]. Taken together, these factors can be conceptualized as early environmental risks (EERs) for mental health development. Prior research has employed EERs as risk factors of attention-deficit/hyperactivity disorder (ADHD), which showed cumulative effects on ADHD symptoms by age 17 [38]. However, few studies have examined these factors together as predictors of co-occurring internalizing and externalizing problems in children [18, 39], and to our knowledge, no study to date has explored how their negative impact changes as children transition into adolescence. The research gaps highlight the need to investigate whether EERs predict later internalizing and externalizing problems and how their influence evolves from childhood to adolescence.

Furthermore, existing studies have highlighted that males and females differ not only in the developmental trajectories of internalizing and externalizing problems [40, 41], but also in their vulnerability to environmental risk factors [42]. In particular, boys are more likely to manifest externalizing behaviors (e.g., aggression, conduct problems) in response to adverse environments, whereas girls tend to show greater susceptibility to internalizing difficulties such as anxiety and depression [42, 43]. These findings underscore the importance of considering potential gender differences when examining both the co-occurrence of internalizing and externalizing problems and the influence of EERs on these symptom domains across development. Through exploring potential gender differences, findings would contribute to early identification of mental health difficulties, and to developing more targeted interventions to mitigate the onset and persistence of symptoms among at-risk individuals [44, 45].

## The present study

To address these research gaps, the current study drew data from a large, population-representative longitudinal study in the UK, tracking individuals from birth to late adolescence. The study has three primary objectives. First, the study investigates the bidirectional associations between internalizing and externalizing problems over time, shedding light on their co-development across key developmental stages. Second, the study examines the long-term effects of early environmental risks, including prenatal factors (i.e., smoking in pregnancy, maternal pre-pregnancy BMI, and blood pressure problems), neonatal factors (i.e., gestation < 37 weeks, birth weight < 2.5 kg, and the absence of breastfeeding), maternal wellbeing (i.e., diagnosis of depression/anxiety, and maternal distress), parenting practices, and socioeconomic status, on internalizing and externalizing problems from ages 5 to 17. Lastly, the study further explores whether these long-term effects of early environmental risks would differ by gender. Findings would help identify critical periods for intervention, informing the timing and focus of targeted strategies aimed at mitigating mental health difficulties throughout childhood and adolescence.

## Methods

### Participants

The sample comprised participants from the UK Millennium Cohort Study (MCS), which is a large, longitudinal, birth cohort including around 19,000 individuals born between 2000 and 2002 [46]. The MCS includes 7 waves of data, starting from 9 months (Wave 1), up to 17 years (Wave 7) of age. The valid sample comprised *N* = 7,377 participants for Model 1 and *N* = 4,930 participants for Model 2.

### Internalizing and externalizing problems

The Strengths and Difficulties Questionnaire (SDQ) [47] was used to measure internalizing and externalizing problems in children. We used the data from Wave 3 (age 5 years), 4 (age 7 years), 5 (age 11 years), 6 (age 14 years), and 7 (age 17 years). Data from ages 5 to 14 years were parent-reported, while age-17 data were self-reported. The internalizing problems were measured by emotional and peer scores (ranging from 0 to 20); externalizing problems were measured via hyperactivity and conduct problems scores (ranging from 0 to 20). Higher scores indicate more severe symptoms. Across all waves used in this study, the SDQ subscales demonstrated adequate internal reliability (see Table S2 for details; [48]).

### Early environmental risks

In line with previous studies [38, 49], early environmental risks (EERs) were grouped into five categories: prenatal risks (i.e., smoking, maternal pre-pregnancy BMI >24.9, and diagnosed hypertension), neonatal risks (i.e., gestation < 37 weeks, birthweight < 2.5 kg, and no breastfeeding), socioeconomic status (i.e., social house/renting from local authority (LA), fewer rooms than people, < 60% median poverty indicator, maternal educational level less than NVQ-3 (i.e., below A-level or equivalent), and only one parent/caregiver), maternal mental health (i.e., diagnosed depression/anxiety and Rutter Malaise Inventory >= 4), and harsh parenting (i.e., smacking, shouting, and telling off). Harsh parenting was measured via three items of the Conflict Tactics Scale [50] and assessed during the second wave sweep (age 3 years), with higher scores indicating a harsher parenting style. The 3-item scale demonstrated acceptable internal reliability (Cronbach’s α = 0.65). The other four EERs were measured at the first wave sweep (9 months), and a cumulative score was calculated for each category. Higher scores represent higher risk. Detailed information of EERs is shown in the supplementary material Table S1.

### Statistical analysis

**Fig. 1:**
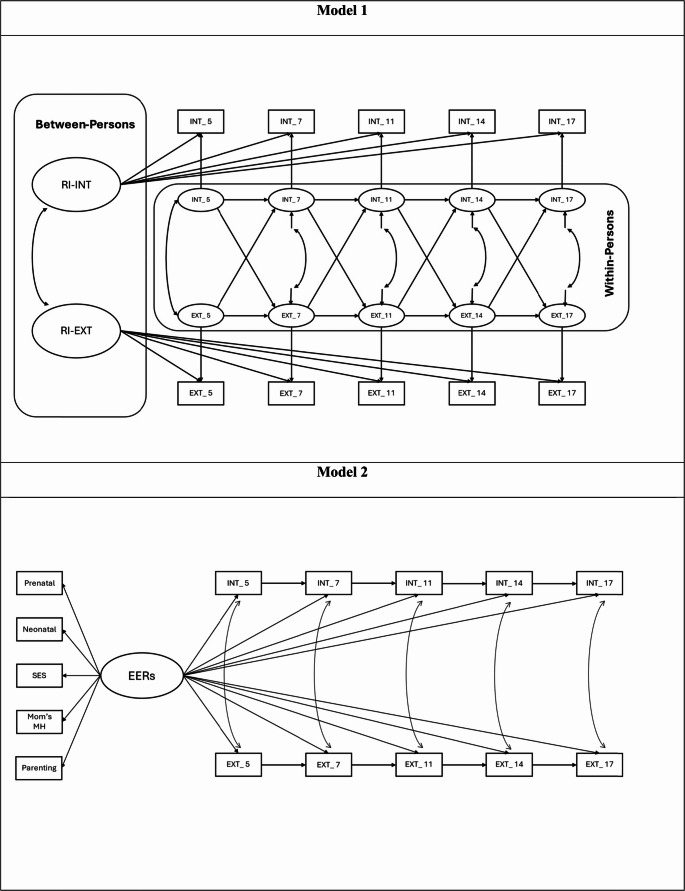
Proposed models

Descriptive and Pearson correlation analyses were conducted on IBM SPSS v29 [51]. A random intercept cross-lagged panel model (RI-CLPM) was used to explore the bidirectional relationship between internalizing and externalizing problems across ages 5 to 17 years (Model 1). Before testing Model 2, a confirmatory factor analysis (CFA) supported the use of early environmental risks (EERs) as a single latent factor (Comparative Fit Index [CFI] = 0.937, Tucker–Lewis Index [TLI] = 0.876, Root Mean Square Error of Approximation [RMSEA] = 0.048, Standardized Root Mean Square Residual [SRMR] = 0.038). Subsequently, we used Structural Equation Modelling (SEM) with a Maximum Likelihood (ML) estimator to test the proposed model (Model 1) and examine the relationship between EERs and later internalizing and externalizing problems from childhood to adolescence (i.e., age 5, 7, 11, 14, and 17 years). Both models were further examined separately for males and females to explore potential sex differences. were conducted in R 4.2.0 [52] with lavaan package [53]. The proposed models (Model 1 and Model 2) are depicted in Fig. 1.

## Results

### Descriptive and correlation

The valid analytic sample for Model 1 was *N* = 7,377 (male = 3458, 46.9%) and for Model 2 was *N* = 4,930 (male = 2407, 48.8%). Detailed descriptive information is shown in Table 1. The correlation results of the variables included in the models are presented in Table 2. Except for the non-significant association between the neonatal score and externalizing problems at age 17 years and that between harsh parenting and internalizing problems at age 17 years, all EERs (i.e., prenatal, neonatal, socioeconomic, maternal mental health, and harsh parenting factor) were significantly and positively associated with both internalizing and externalizing problems at all subsequent time points (i.e., age 5, 7, 11, 14, and 17 years).

**Table 1:** Descriptive information

	Variables	*N* (%)	Mean (SD)	Range
Sex	Male	3458 (46.9%)		
	Female	3700 (50.2%)		
	Missing	219 (3.0%)		
Ethnicity	White	6265 (84.9%)		
	Mixed	184 (2.5%)		
	Indian	167 (2.3%)		
	Pakistani and Bangladeshi	306 (4.1%)		
	Black or Black British	158 (2.1%)		
	Other Ethnic group	66 (0.9%)		
	Missing	231 (3.1%)		
Internalizing Problems	Wave 3		2.36 (2.41)	0–20
	Wave 4		2.53 (2.64)	0–20
	Wave 5		3.02 (3.04)	0–20
	Wave 6		3.55 (3.33)	0–20
	Wave 7		5.67 (3.51)	0–20
Externalizing Problems	Wave 3		4.44 (3.25)	0–20
	Wave 4		4.35 (3.44)	0–20
	Wave 5		4.11 (3.38)	0–20
	Wave 6		3.98 (3.37)	0–20
	Wave 7		5.61 (3.29)	0–20
Early Environment Risk	Prenatal Score		0.51 (0.65)	0–3
(*N* = 4930)	Neonatal Score		0.32 (0.58)	0–3
	SES Score		0.78 (1.05)	0–5
	Maternal Mental Health Score		0.33 (0.59)	0–2
	Harsh Parenting Score		9.48 (2.27)	3–15

**Table 2:** Correlation between used variables

	Prenatal	Neonatal	SES	Maternal Mental	Parenting	SDQ_INT_W3	SDQ_EXT_W3	SDQ_INT_W4	SDQ_EXT_W4	SDQ_INT_W5	SDQ_EXT_W5	SDQ_INT_W6	SDQ_EXT_W6	SDQ_INT_W7	SDQ_EXT_W7
Prenatal	1	0.114^***^	0.218^***^	0.130^***^	0.053^***^	0.088^***^	0.155^***^	0.070^***^	0.145^***^	0.105^***^	0.150^***^	0.124^***^	0.138^***^	0.093^***^	0.048^**^
Neonatal		1	0.216^***^	0.041^**^	−0.015	0.074^***^	0.100^***^	0.078^***^	0.094^***^	0.072^***^	0.080^***^	0.076^***^	0.078^***^	0.051^***^	0.023
SES			1	0.163^***^	−0.001	0.156^***^	0.214^***^	0.166^***^	0.205^***^	0.173^***^	0.222^***^	0.186^***^	0.202^***^	0.114^***^	0.080^***^
Maternal Mental				1	0.092^***^	0.166^***^	0.167^***^	0.170^***^	0.160^***^	0.182^***^	0.162^***^	0.192^***^	0.136^***^	0.127^***^	0.067^***^
Parenting					1	0.089^***^	0.287^***^	0.094^***^	0.245^***^	0.118^***^	0.256^***^	0.090^***^	0.197^***^	− 0.030^*^	0.081^***^
SDQ_INT_W3						1	0.341^***^	0.587^***^	0.287^***^	0.460^***^	0.265^***^	0.393^***^	0.231^***^	0.155^***^	0.067^***^
SDQ_EXT_W3							1	0.320^***^	0.715^***^	0.334^***^	0.639^***^	0.308^***^	0.541^***^	0.092^***^	0.243^***^
SDQ_INT_W4								1	0.396^***^	0.569^***^	0.330^***^	0.464^***^	0.280^***^	0.176^***^	0.102^***^
SDQ_EXT_W4									1	0.370^***^	0.714^***^	0.326^***^	0.605^***^	0.096^***^	0.272^***^
SDQ_INT_W5										1	0.452^***^	0.618^***^	0.359^***^	0.263^***^	0.135^***^
SDQ_EXT_W5											1	0.376^***^	0.720^***^	0.123^***^	0.316^***^
SDQ_INT_W6												1	0.444^***^	0.354^***^	0.145^***^
SDQ_EXT_W6													1	0.127^***^	0.372^***^
SDQ_INT_W7														1	0.328^***^
SDQ_EXT_W7															1

Note. *N* = 4,930; *p <.05; ** *p* <.01; *** *p* <.001

### Longitudinal relationship between internalizing and externalizing problems

**Fig. 2:**
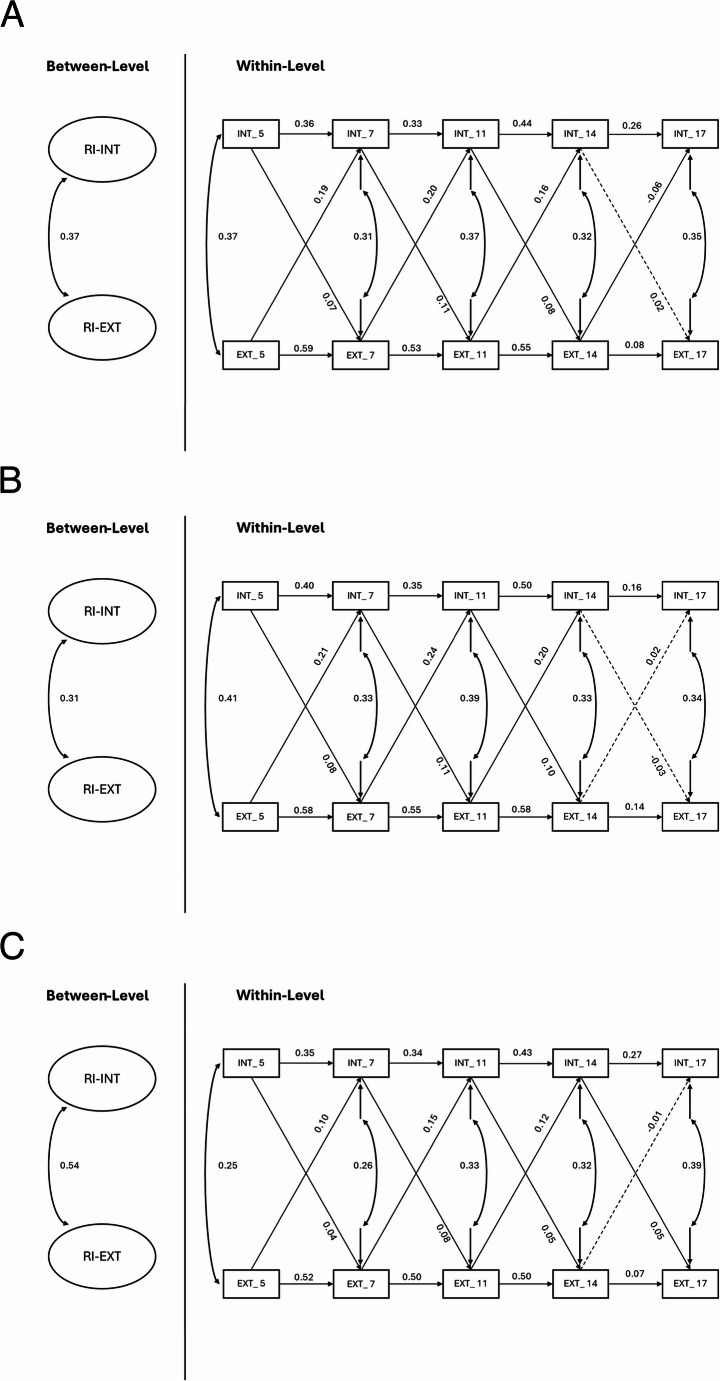
**a** Detailed results of the Random Intercept Cross-Lagged Panel Model (*N* = 7,377). **b** Detailed results from the Random Intercept Cross-Lagged Panel Model for males (*N* = 3,458). **c** Detailed results from the Random Intercept Cross-Lagged Panel Model for females (*N* = 3,700). Note. Standardized path coefficients are reported; INT = Internalizing problems; EXT = Externalizing problems; The numbers correspond to the age of the index child at the assessment time (e.g., INT_5 refers to internalizing problems measured at 5 years of age); Solid lines represent significant results; Dashed lines represent non-significant results

The RI-CLPM model showed a good model fit (see Fig. 2a; CFI = 0.977; TLI = 0.952; RMSEA = 0.069; SRMR = 0.047). For bidirectional associations, internalizing problems at ages 5, 7, and 11 positively and significantly predicted externalizing problems at ages 7 (*β* = 0.07, *p* <.001), 11 (*β* = 0.11, *p* <.001), and 14 (*β* = 0.08, *p* <.001), respectively. Similarly, externalizing problems at ages 5, 7, and 11 positively and significantly predicted internalizing problems at ages 7 (*β* = 0.19, *p* <.001), 11 (*β* = 0.20, *p* <.001), and 14 (*β* = 0.16, *p* <.001), respectively. The same path between ages 14 to 17 was negative (*β* = −0.06, *p* <.001). No significant results were found between internalizing problems at age 14 and externalizing problems at age 17. The random intercepts for internalizing problems and externalizing problems showed a significant positive association (*β* = 0.37, *p* <.001), indicating participants with higher stable levels of internalizing problems also tended to exhibit higher stable levels of externalizing problems. At the within-person level, significant positive associations were found between internalizing and externalizing problems across waves (*β* = 0.25–0.39, all *p* <.001).

For males (see Fig. 2b; CFI = 0.975; TLI = 0.947; RMSEA = 0.074; SRMR = 0.049; *N* = 3,458), internalizing problems at ages 5, 7, and 11 significantly predicted externalizing problems at the subsequent waves (β = 0.08–0.11, all *p* <.001), while externalizing problems at the same ages significantly predicted subsequent internalizing problems (β = 0.20–0.24, all *p* <.001). However, neither internalizing nor externalizing problems at age 14 significantly predicted the other at age 17.

For females (see Fig. 2c; CFI = 0.978; TLI = 0.953; RMSEA = 0.065; SRMR = 0.048; *N* = 3,700), internalizing problems at ages 5, 7, 11, and 14 significantly predicted externalizing problems at the subsequent waves (β = 0.04–0.08, all *p* <.01), while externalizing problems also significantly predicted subsequent internalizing problems (β = 0.10–0.15, all *p* <.05) except the path from age 14 to 17.

The correlation between the random intercepts was positive and significant for both males (β = 0.31; *p* <.001) and females (β = 0.54; *p* <.001). Within-person concurrent associations between internalizing and externalizing problems were positive and significant across all waves for both males (β = 0.33–0.41, all *p* <.001) and females (β = 0.25–0.39, all *p* <.001).

### Early environmental risks (EERs) and internalizing/externalizing problems

**Fig. 3:**
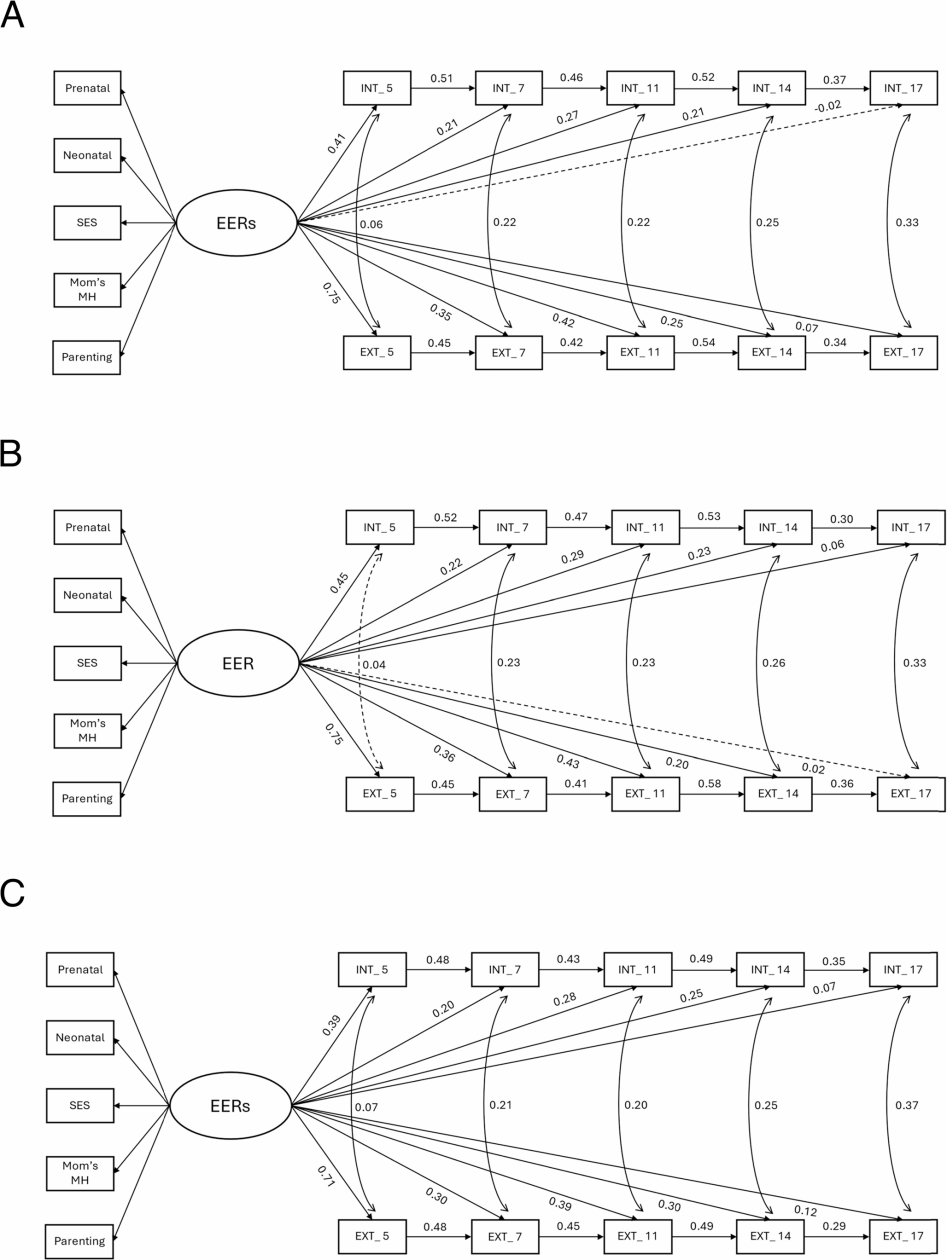
**a** Detailed results from Model 2 for the whole sample (*N* = 4,930). **b** Detailed results from Model 2 for males (*N* = 2,407). **c** Detailed results from Model 2 for females (*N* = 2,523). Note. Standardized path coefficients are reported; EERs = Early environmental risks; INT = Internalizing problems; EXT = Externalizing problems; The numbers correspond to the age of the index child at the assessment time (e.g., INT_5 refers to internalizing problems measured at 5 years of age); Solid lines represent significant results; Dashed lines represent non-significant results

Figure 3a shows the results of Model 2 (CFI = 0.957; TLI = 0.942; RMSEA = 0.052; SRMR = 0.040; *N* = 4,930), testing associations between EERs and later mental health conditions via Structural Equation Modelling. The results show that EERs positively and significantly predict later internalizing problems at ages 5 (*β* = 0.41, *p* <.001), 7 (*β* = 0.21, *p* <.001), 11 (*β* = 0.27, *p* <.001), and 14 (*β* = 0.21, *p* <.001). Externalizing problems at ages 5 (*β* = 0.75, *p* <.001), 7 (*β* = 0.35, *p* <.001), 11 (*β* = 0.42, *p* <.001), 14 (*β* = 0.25, *p* <.001), and 17 (*β* = 0.07, *p* <.001) were also positively and significantly predicted by EERs. The autoregressive paths of both internalizing and externalizing problems from ages 3 to 17 years were positive and significant (all *p* <.001), indicating that an individual’s internalizing and externalizing problems in a given wave were strongly predicted by their levels in the previous wave. Additionally, the covariances between internalizing and externalizing problems were positive and significant across all waves, suggesting a stable and potentially strengthening relationship between the two as children age.

For males (see Fig. 3b; CFI = 0.957; TLI = 0.942; RMSEA = 0.053; SRMR = 0.038; *N* = 2,407), EERs significantly predicted internalizing problems at all ages from 5 to 17 (β = 0.06–0.45, all *p* <.05) and externalizing problems at ages from 5 to 14 (β = 0.20–0.75, all *p* <.001). Autoregressive paths for both internalizing and externalizing problems remained positive and significant across waves (all *p* <.001). Concurrent associations between the two domains were positive and significant at all waves except age 5 (*p* =.12).

For females (see Fig. 3c; CFI = 0.961; TLI = 0.947; RMSEA = 0.048; SRMR = 0.037; *N* = 2,523), EERs significantly predicted both internalizing problems (β = 0.07–0.39, all *p* <.01) and externalizing problems at all ages from 5 to 17 (β = 0.12–0.71, all *p* <.001). Autoregressive paths for both internalizing and externalizing problems (all *p* <.001), as well as concurrent associations between the two domains, were positive and significant across waves (all *p* <.05).

## Discussion

To our knowledge, this is the first study to examine the long-term effects of a range of early environmental risk factors on subsequent internalizing and externalizing problems, as well as the bidirectional relationship between internalizing and externalizing problems from early childhood to late adolescence. By situating the analysis within the framework of early environmental risks (EERs), the study provides a comprehensive, longitudinal perspective on how early-life adversity shapes mental health trajectories over time. Existing research has explored the bidirectional association between internalizing and externalizing problems within distinct developmental stages. However, findings regarding the directionality of this relationship are inconsistent (e.g., [2, 15, 16]). The current study extends previous research by moving the focus beyond separate developmental stages and examining the co-development of internalizing and externalizing problems from early childhood to late adolescence. By adopting a longitudinal approach, we provide a more comprehensive understanding of how these mental health problems evolve over an extended period. In addition, while prior studies have identified various early life risk factors for adverse mental health outcomes, few have integrated these factors within an EER framework to examine their lasting impact on developmental trajectories. The present study addresses this knowledge gap by considering multiple dimensions of environmental risk, including prenatal influences, neonatal factors, maternal mental health, harsh parenting, and socioeconomic status, highlighting the enduring effects of early adversity on child and adolescent mental health. The findings underscore the critical role of early intervention and prevention in mitigating risk factors within family and environmental contexts.

Through adopting the RI-CLPM approach, our findings provide insight into the co-occurrence and developmental trajectories of internalizing and externalizing problems. The analyses showed three notable patterns. First, at the between-person level, internalizing and externalizing were positively associated, suggesting that individuals with chronically higher internalizing symptoms also tended to report higher externalizing symptoms across the study period. Second, within individuals, the substantial autoregressive effects indicated high stability of both problem domains over time, suggesting that early manifestations of mental ill-health (i.e., internalizing and externalizing problems) tend to persist through childhood and adolescence. These findings are consistent with prior research demonstrating the long-term stability of internalizing and externalizing problems [15, 54]. Third, significant bidirectional associations were observed between internalizing and externalizing problems at earlier developmental stages. Specifically, internalizing and externalizing problems at ages 5, 7, and 11 each positively predicted the other type of problem in subsequent waves. These findings are consistent with previous studies [28, 55, 56] and support the adjustment erosion hypothesis [14], according to which difficulties in one domain can undermine overall adjustment, leading to cascading effects on the other domain over time.

However, the pattern of associations changed in late adolescence. Internalizing problems at age 14 did not significantly predict externalizing problems at age 17, while externalizing problems at age 14 were negatively associated with internalizing problems at age 17. These findings suggest a shift in the nature of the relationship between these mental health problems during mid-to-late adolescence. One possible explanation is that engagement in externalizing behaviors, such as risk-taking or aggression, during mid-adolescence may serve as a coping mechanism, temporarily alleviating negative emotions and reducing subsequent internalizing problems [7, 57]. The reinforcement perspective further proposes that externalizing behaviors may function as an adaptive response to environmental stressors [58], providing adolescents with a sense of control or immediate reward, thereby mitigating internalizing symptoms. Another plausible explanation lies in the informant effect [59], which is related to the measurement differences at the different assessment times. Specifically, in the first four waves, internalizing and externalizing problems were assessed through parental reports, whereas at age 17, adolescents’ self-reports were used. A recent study based on the same dataset as the present study has revealed that adolescents tend to report more negatively and less positively on the SDQ scales compared to parental reports [60]. Therefore, the discrepancy between parent and adolescent perceptions of mental health problems may contribute to the observed inconsistency in directionality [59, 61].

Regarding gender differences in the RI-CLPM, for both males and females, internalizing and externalizing problems were significantly positively associated at the between-individual level, with the two domains being more tightly intertwined for females than for males. The within-person dynamics revealed developmental and gender-specific patterns. In the male group, bidirectional associations were observed only during early and middle childhood, whereas these cross-lagged effects were not significant from age 14 to 17. The results suggested that the reciprocal influence of the two domains may diminish as males transition into late adolescence. In contrast, for females, reciprocal cross-lagged associations were observed more consistently across ages 5 through 14. Only the pathway from externalizing at age 14 to internalizing at age 17 failed to reach significance. These findings suggest that the interplay between internalizing and externalizing problems is more sustained for girls than for boys across developmental stages.

Moving forward, the bidirectional relationship between internalizing and externalizing problems observed in the whole sample, as well as the gender subsamples, may arise from common underlying mechanisms shaped by shared risk factors. Building on this, the study further examined whether early environmental risk factors have long-term effects on both internalizing and externalizing problems. Our findings support the shared risk hypothesis [16]. Specifically, EERs positively predicted internalizing problems from early childhood to early adolescence, and externalizing problems from early childhood to late adolescence. Notably, the strength of these predictive effects generally declined with age, aligning with previous literature suggesting that the influence of early environmental risks on mental health weakens during adolescence [62]. However, an exception to this trend emerged at age 11, where a temporary increase in the predictive effect was observed. This spike may reflect the developmental transitions and environmental stressors that typically occur in late childhood, such as school changes and increasing social demands [63]. Children exposed to early environmental risks often have fewer resources to navigate these challenges and transitions, and this, in turn, may amplify the impact of early adversity on both internalizing and externalizing problems [63]. As individuals transition through adolescence and begin to gain greater autonomy, they are increasingly influenced by proximal and concurrent risk factors, such as peer relationships [64, 65]. This shift in influence may help explain the gradual weakening of early environmental risk effects over time, reinforcing the dynamic nature of risk exposure and adaptation in adolescent mental health. In addition, when the effects of EERs were incorporated into the model, the covariance between internalizing and externalizing problems showed a slight increasing trend from age 5 through age 17. This pattern indicates that early environmental risks may contribute not only to elevated levels of each symptom domain, but also to their co-occurrence across development. Such findings lend further support to the shared risk hypothesis, suggesting that EERs shape both internalizing and externalizing trajectories simultaneously and thereby heighten their developmental interdependence.

The enduring effects of EERs on both internalizing and externalizing problems also exhibited variation by gender. Among males, EERs predicted elevated internalizing symptoms consistently across all ages, while the impact on externalizing problems was significant through early to mid-adolescence but diminished by age 17. In contrast, for females, EERs significantly predicted both internalizing and externalizing problems across all developmental stages examined, indicating more persistent effects of early adversity. These findings reveal gender differences in the long-term effects. For females, the persistence of the effects of EERs on both symptom domains suggests that females may be more susceptible to the lasting effects of early adversity, particularly due to mechanisms such as higher female sensitivity and internalization of distress [42]. In contrast, the effects of EERs on male externalizing problems diminish in late adolescence, consistent with prior literature suggesting that externalizing problems typically peak in late childhood and early adolescence, then decline as boys mature cognitively, develop greater impulse control, and encounter stronger social constraints in late adolescence [66].

This study has several limitations warranting acknowledgment. First, although this study aimed to provide a comprehensive examination of early environmental risk in relation to subsequent child and adolescent mental health, some relevant factors (e.g., parental substance use, paternal mental health, marital conflict) were not included due to data availability [24, 67–70]. In addition, the current study only focused on environmental factors assessed during the earliest developmental periods, specifically infancy (9 months) and toddlerhood (age 3). Nonetheless, additional risks may emerge beyond these periods. For example, later and concurrent environmental factors across ecological systems (e.g., child maltreatment, peer victimization, school climate, and collective efficacy) may also exert prominent effects on both internalizing and externalizing problems across development [71, 72]. Therefore, future research should adopt a more comprehensive life-course approach that incorporates both early and subsequent exposures to understand how the timing and accumulation of risk contribute to mental health trajectories. Second, the study sample was predominantly White (84.9%), which limits the generalizability of the findings to more diverse populations. Future research should therefore replicate these findings in more diverse samples to enhance external validity. A further central limitation related to the lack of diversity in the study sample lies in genetic influences, as gene-environment interplay may vary across ethnic and cultural groups. Due to data unavailability, the current study did not consider the impact of genetic factors on internalizing and externalizing problems. Given the well-established role of genetic predispositions in internalizing and externalizing problems [73], future research should integrate genetic data to examine the interplay between genetic vulnerability and early environmental risks in shaping adolescent mental health [17, 21]. Third, this study considered internalizing and externalizing as broad constructs. However, the internalizing domain comprises both emotional symptoms (e.g., anxiety and depressive symptoms) and peer problems, while the externalizing domain includes conduct problems (e.g., aggression, rule-breaking) and hyperactivity/inattention. Future research should disaggregate these domains to explore how EERs might differentially influence distinct specific symptom dimensions at various stages of development, using assessment measures that allow this disaggregation. Fourth, the reliance on parental reports for most key variables (EER and internalizing and externalizing outcomes from ages 5 to 14) raises concerns about potential reporter bias and inflated associations due to shared method variance [18]. While parents are valuable informants and parent reports have been widely used in developmental research [74], their perceptions may not fully capture the adolescent’s internal experiences. Future studies should adopt multi-informant approaches (e.g., parents, teachers, and self-reports) to enhance the validity and reliability of findings [75]. Finally, the current study assessed internalizing and externalizing problems using parent-reported SDQ from ages 5 to 14, whereas adolescent self-reported SDQ was used at age 17, primarily due to the limited measurement invariance of the parent-reported SDQ at this stage. Previous studies have adopted a similar approach by using adolescent self-reports at age 17 in the MCS, which demonstrated acceptable internal reliability [48, 60]. Moreover, research has suggested that self-reports may provide a more accurate assessment of mental health in late adolescence, as adolescents gain greater independence and capacity to report their mental health symptoms [48, 76]. Nonetheless, the change in informants may have contributed to the different patterns observed in the cross-lagged effects from ages 14 to 17, as well as the varied long-term effects of EERs on both domains. Future studies should therefore employ consistent informants across developmental stages to improve comparability and reduce potential biases in longitudinal assessments of internalizing and externalizing problems. In addition, a systematic comparison of parent- and self-reported mental health symptoms would help to disentangle whether the observed differences are primarily attributable to informant effects or reflect changes in the frequency or severity of symptoms.

Notwithstanding these limitations, the current study offers important theoretical and practical contributions. By utilizing data from a large, nationally representative UK cohort, our findings can be generalized to a broader population. Furthermore, the study’s comprehensive measurement of EER (most factors assessed at birth, except for harsh parenting, which was measured at age 3) provides valuable insights into how early (i.e., before age 3) environmental adversity exerts enduring impacts on individuals’ mental health spanning from early childhood to late adolescence. The findings emphasize the importance of adopting a developmental perspective when assessing the impact of early risk factors. Furthermore, the longitudinal data allowed us to obtain a comprehensive overview of the co-development of internalizing and externalizing problems across key developmental stages.

From a practical standpoint, these results highlight the critical need for early identification of at-risk families and early intervention. Given the lasting impact of EER, health and social services should prioritize comprehensive assessments to identify at-risk families and implement timely targeted interventions that address maternal mental health and family functioning. In addition, the co-occurrence and high stability of internalizing and externalizing problems underscore the necessity for integrated, longitudinal mental health interventions. Considering that internalizing and externalizing problem domains may reinforce each other over time, early detection of one problem should prompt concurrent assessment and intervention of the other. Moreover, the different strengths of the relationship at different time points suggest that intervention strategies should be developmentally tailored. For example, efforts to disrupt the cycle of co-occurring problems should focus on middle childhood and early adolescence, while interventions targeting late adolescence should account for the shifting nature of internalizing and externalizing problem interactions. By providing an understanding of the interplay between early environmental risks and adolescent mental health trajectories, this study informs both research and clinical practice, reinforcing the importance of early and developmentally informed interventions.

## Conclusion

Using seven waves of data from a large, nationally representative UK-based cohort, this study highlights the dual nature of mental health problems: while both internalizing and externalizing symptoms show considerable stability over time, their bidirectional relationship evolves, with shifts in strength and direction across developmental stages. Furthermore, the findings demonstrate that early environmental risk factors exert a lasting influence on child and adolescent mental health, shaping the developmental trajectories of internalizing and externalizing problems from early childhood to late adolescence. Notably, the transition from early to late adolescence marks a critical period of change, suggesting that mental health challenges are not static but dynamically influenced by individual development and environmental contexts. These findings underscore the need for prevention and intervention programs that are both early and sustained, addressing risk factors comprehensively while adapting to the evolving nature of adolescent mental health.

## Supplementary Information

Below is the link to the electronic supplementary material.

Supplementary Material 1 (DOCX 19.3 KB)

## Data Availability

All datasets used for this study are freely available to researchers in the UK via the UK Data Service (https://ukdataservice.ac.uk).
