# Supplementary material for: Early environmental risks and the developmental dynamics of internalizing and externalizing problems from birth to adolescence
Source: Eur Child Adolesc Psychiatry. 2025 Nov 6;35(3):1007–20. doi: 10.1007/s00787-025-02887-3 (PMC13212713; doi:10.1007/s00787-025-02887-3)
Supplement: Supplementary file 1 — Supplementary Material 1 (DOCX 19.3 KB) [file 787_2025_2887_MOESM1_ESM.docx]

Table S1. Detailed information about early environmental risks

|  | **Assessment time** | **Cut-off for high-risk** |
| --- | --- | --- |
| **Prenatal Risks** | 9 Months (Wave 1) | Smoking in pregnancy |
|  | 9 Months (Wave 1) | Maternal pre-pregnancy BMI>24.9 |
|  | 9 Months (Wave 1) | Antenatal blood pressure |
|  |  |  |
| **Neonatal Risks** | 9 Months (Wave 1) | Gestation<37 weeks |
|  | 9 Months (Wave 1) | Birthweight <2.5kg |
|  | 9 Months (Wave 1) | Non Breastfeeding |
|  |  |  |
| **Socioeconomic**  **Status Risks** | 9 Months (Wave 1) | Housing tenure (Social House/ Renting from LA) |
|  | 9 Months (Wave 1) | Household crowding (People>rooms) |
|  | 9 Months (Wave 1) | Household income (Below 60% median poverty indicator) |
|  | 9 Months (Wave 1) | Maternal educational level (NVQ<3: level below A-level or its equivalent) |
|  | 9 Months (Wave 1) | One parent/caregiver |
|  |  |  |
| **Maternal Mental Health Risks** | 9 Months (Wave 1) | Depression/anxiety |
|  | 9 Months (Wave 1) | Maternal distress (RMI>=4) |
|  |  |  |
| **Parenting Risks** | Age 3 (Wave 2) | Smacking |
|  | Age 3 (Wave 2) | Shouting |
|  | Age 3 (Wave 2) | Telling off |

**Table S2. Internal reliability for SDQ subscales across study waves**

|  | Age | EMO | CB | Hyper | Peer | Pro |
| --- | --- | --- | --- | --- | --- | --- |
| SDQ W3 | 5 | 0.604 | 0.566 | 0.747 | 0.436 | 0.63 |
| SDQ W4 | 7 | 0.652 | 0.602 | 0.789 | 0.576 | 0.699 |
| SDQ W5 | 11 | 0.708 | 0.633 | 0.782 | 0.594 | 0.654 |
| SDQ W6 | 14 | 0.724 | 0.647 | 0.775 | 0.623 | 0.736 |
| SDQ W7 | 17 | 0.741 | 0.56 | 0.727 | 0.556 | 0.66 |

**Note.** Cronbach’s alpha was used to measure internal reliability; EMO=emotional problems; CB=conduct behaviors; Hyper=hyperactivity/inattention subscales; Peer=peer problems; Pro=prosocial behaviors.
